# Late presentation of extrauterine adenomyomas after laparoscopic morcellation at hysterectomy: a case report

**DOI:** 10.1186/s12905-021-01408-z

**Published:** 2021-07-07

**Authors:** Mohammed Nagdi Zaki, Aafia Mohammed Farooq Gheewale, Nada Ibrahim, Ibrahim Abd Elrahman

**Affiliations:** 1 College of Medicine, Mohammed Bin Rashid University of Medicine and Health Sciences, Dubai Healthcare City, Building 14, 505055 Dubai, United Arab Emirates; 2 Department of Pathology and Laboratory Medicine, Mediclinic City Hospital, Dubai Healthcare City, Building 37, 505004 Dubai, United Arab Emirates; 3 Department of Obstetrics and Gynecology, Mediclinic City Hospital, Dubai Healthcare City, Building 37, 505004 Dubai, United Arab Emirates

**Keywords:** Extrauterine adenomyoma, Laparoscopic hysterectomy, Morcellation, Case report

## Abstract

**Background:**

An adenomyoma is a well circumscribed form of adenomyosis and can be located within the myometrium, in the endometrium as a polyp, or extrauterine with the last being the rarest presentation amongst the three. With the ongoing advancement in gynecological surgery, the use of electromechanical morcellators have made the removal of large and dense specimens possible with minimally invasive techniques. However, it has also caused an increase in complications which were previously rare. Whilst the tissue is being grinded within the abdominal cavity, residual tissue can spread and remain inside, allowing for implantation to occur and thereby giving rise to recurrence of uterine tissue as a new late postoperative complication.

Case presentation

A 45-year-old woman presented with worsening constipation and right iliac fossa pain. Her past surgical history consists of laparoscopic supra-cervical hysterectomy that was indicated due to uterine fibroids. Computerized tomography and magnetic resonance imaging were done, which showed an irregular lobulated heterogeneous mass seen in the presacral space to the right, located on the right lateral aspect of the recto-sigmoid, measuring 4.5 × 4.3 × 4.3 cm in size. A transvaginal ultrasound revealed a cyst in the left ovary. The patient had a treatment course over several months that included Dienogest (progestin) and Goserelin (GnRH analogue) with add-back therapy. In line with the declining response to medications, the patient was advised for a laparoscopic ovarian cystectomy. During the surgery, an additional lesion was found as a suspected fibroid and the left ovarian cyst was identified as pockets of peritoneal fluid which was sent for cytology. The surgical pathology report confirmed adenomyosis in both specimens, namely the right mass and the initially suspected fibroid.

**Conclusion:**

In this case report, we showcase a rare occurrence of an extrauterine adenomyoma presenting two years post laparoscopic morcellation at hysterectomy. This poses questions regarding the benefits versus risks of power morcellation in laparoscopic hysterectomy.

## Background

An adenomyoma is a well circumscribed form of adenomyosis. It is a nodular aggregate that has the properties of smooth muscle, endometrial glands and endometrial stroma. It may be located within the myometrium, in the endometrium as a polyp or exist as an extrauterine adenomyoma. Extrauterine adenomyomas are the rarest presentation amongst the three [1]. While the development of adenomyosis is poorly understood, the most credible theory is an atypical invagination of the basalis layer of the endometrium into the adjoining myometrial layer. Hypothetical causal factors for the invagination to occur consist of mechanical disturbance of the endometrial-myometrial interface, hormonal imbalance, and impaired immunity [2]. Due to the diagnosis of adenomyosis being solely dependent on histological analysis, an accurate presentation of its incidence or prevalence is challenging, leading to a variable set of estimates with a range of 5% to 70% being that of its prevalence [3].

With the ongoing advancement in gynecological surgery, multiple options have taken lead in yielding minimally invasive surgical options, those inclusive of electromechanical morcellator, laparoscopic myomectomy and laparoscopic hysterectomy. Additionally, conservative options include high intensity focused ultrasound (HIFU) which involves using great ultrasound energy targeted at abnormal tissues and their vascularity directly. This is done via heating and cavitation exempting the surrounding normal tissue of any damage. This process can be guided via magnetic resonance imaging (MRgHIFU) or via ultrasound (USgHIFU) [4]. Another conservative method of treatment called uterine artery embolization (UAE) exists, which is indicated for symptomatic uterine fibroids. It is carried out by directing transarterial catheters into the uterine artery and its branches under fluoroscopy to induce necrosis within the adenomyotic tissue [4]. While the use of electromechanical morcellators has made the removal of large and dense specimens like a leiomyoma convenient and minimally invasive, it has also brought forth a rise in complications. In addition to the standard existing postoperative complications such as fever, bladder injury, hemorrhage, etc. which mark an incidence of 1.65–4% according to literature, newer complications have arose [5]. Due to the limitations of the laparoscopic technique, recurrence of uterine tissue as a new late postoperative complication can take place. This is because of small residues of endometrial tissue fragments that could remain in the abdominal cavity during the removal of uterine tissue. Herein, we report a rare case of an extrauterine adenomyoma presenting two years after a laparoscopic hysterectomy which was accurately diagnosed by histopathology.

## Case presentation

A 45-year-old woman presented with worsening constipation and right iliac fossa pain in late October 2019. She had a similar episode in May 2019 that required admission. Her past surgical history consists of laparoscopic supracervical hysterectomy which was indicated due to uterine fibroids. The surgery was done in November 2017. The patient also has a history of immune thrombocytopenia, autoimmune hemolytic anemia—Evan’s Syndrome, iron deficiency anemia as well as hypertriglyceridemia. The serum levels of CA125 and Ca19-9 were 169 U/mL and 36 U/mL respectively. CT and MRI showed an irregular lobulated heterogeneous signal intensity mass seen in the presacral space to the right, located on the right lateral aspect of the recto-sigmoid measuring 4.5 × 4.3 × 4.3 cm in size. The mass showed heterogeneous enhancement with areas of non-enhancing cystic component. The mass was abutting the right piriformis muscle and right anterior aspect of the rectum. A right internal iliac enhancing node was seen measuring 1.1 × 0.8 cm in size with fluid in the pouch of Douglas. The uterocervical stump and both ovaries appeared normal. This was followed by a colonoscopy that revealed no abnormal findings. A CT guided fine needle biopsy of the pelvic mass was done, that revealed endometriosis with microscopic findings of focal areas of benign endometrial epithelium, and stroma involving smooth muscle tissue on histology with no morphological features of dysplasia or malignancy.

**Fig. 1:**
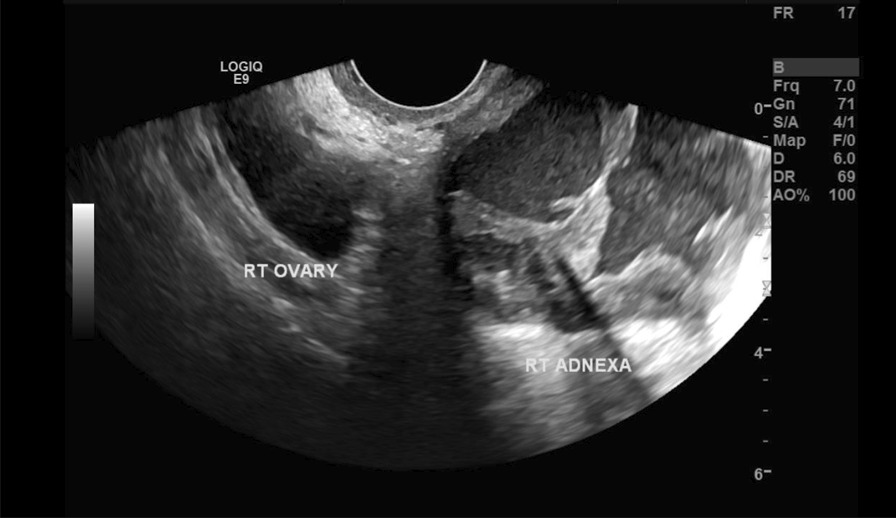
Ultrasound reveals right sided mass. The mass appears irregularly lobulated and heterogeneous measuring 4.5 × 4.3 × 4.3 cm in size

**Fig. 2:**
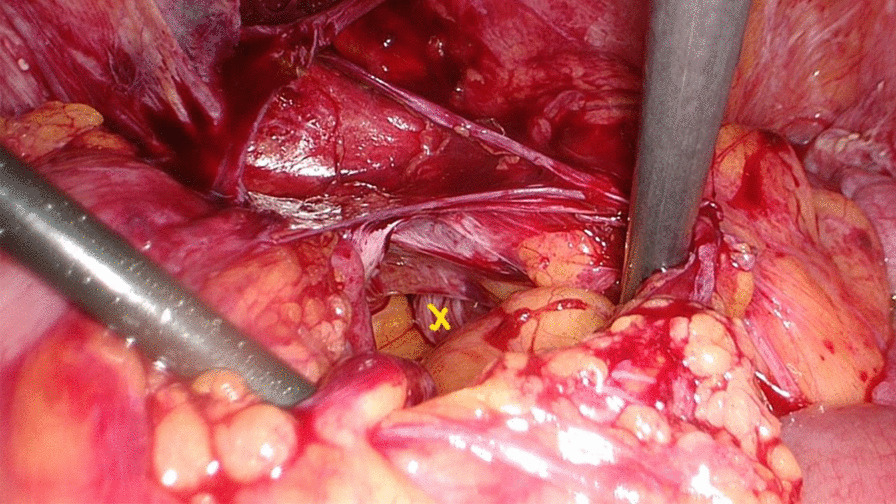
Right mass buried in Pouch of Douglas. Extensive adhesiolysis was required to access the mass entirely

**Fig. 3:**
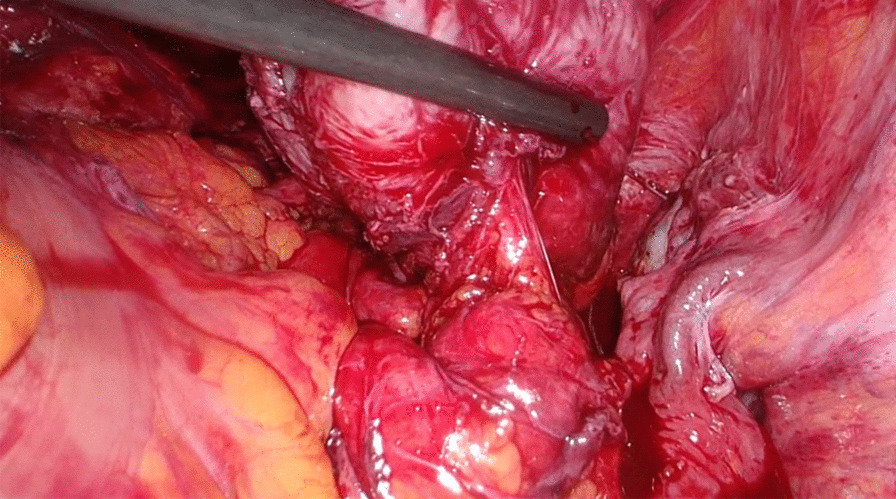
Right mass adherent to sigmoid colon. As shown in the CT and MRI, the mass was found in the presacral space and on the right lateral aspect of the recto-sigmoid colon

**Fig. 4:**
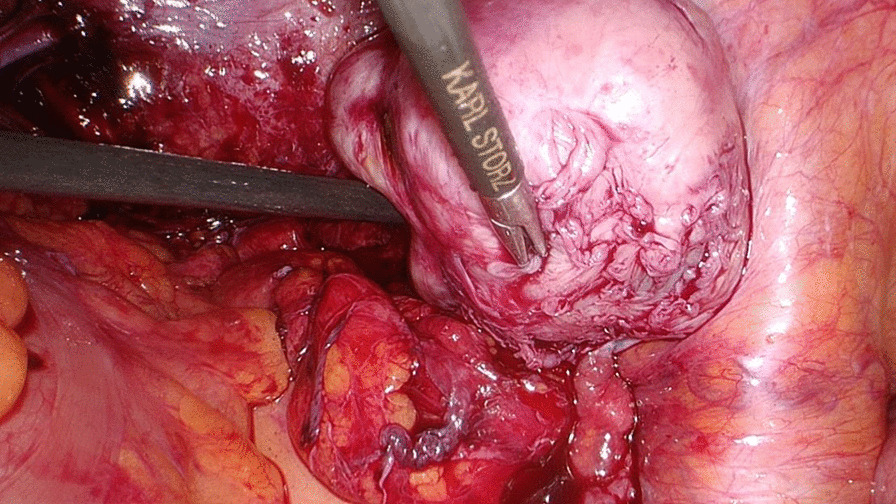
Post adhesiolysis and mobilizing the right mass. This mass was sent for histopathology and was confirmed to be an adenomyoma

**Fig. 5:**
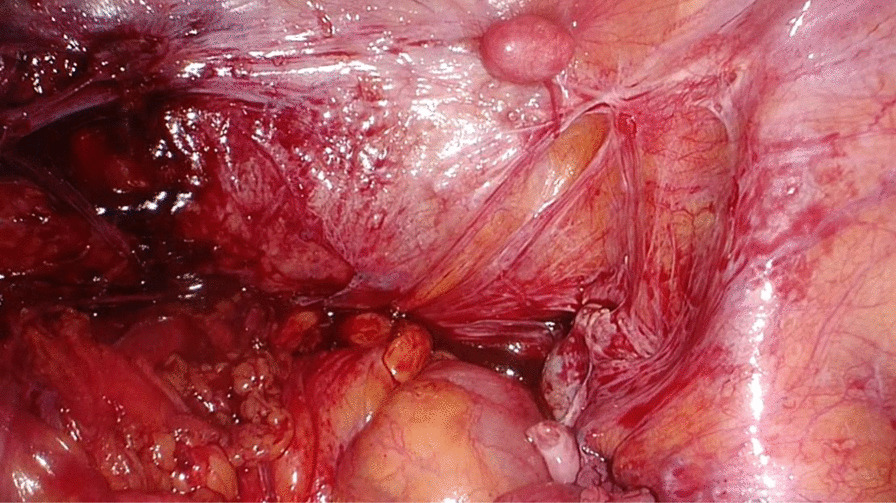
Suspected uterine fibroid. This 1 cm mass was found incidentally intraoperatively and was sent for histopathology, which confirmed it to be an adenomyoma

**Fig. 6:**
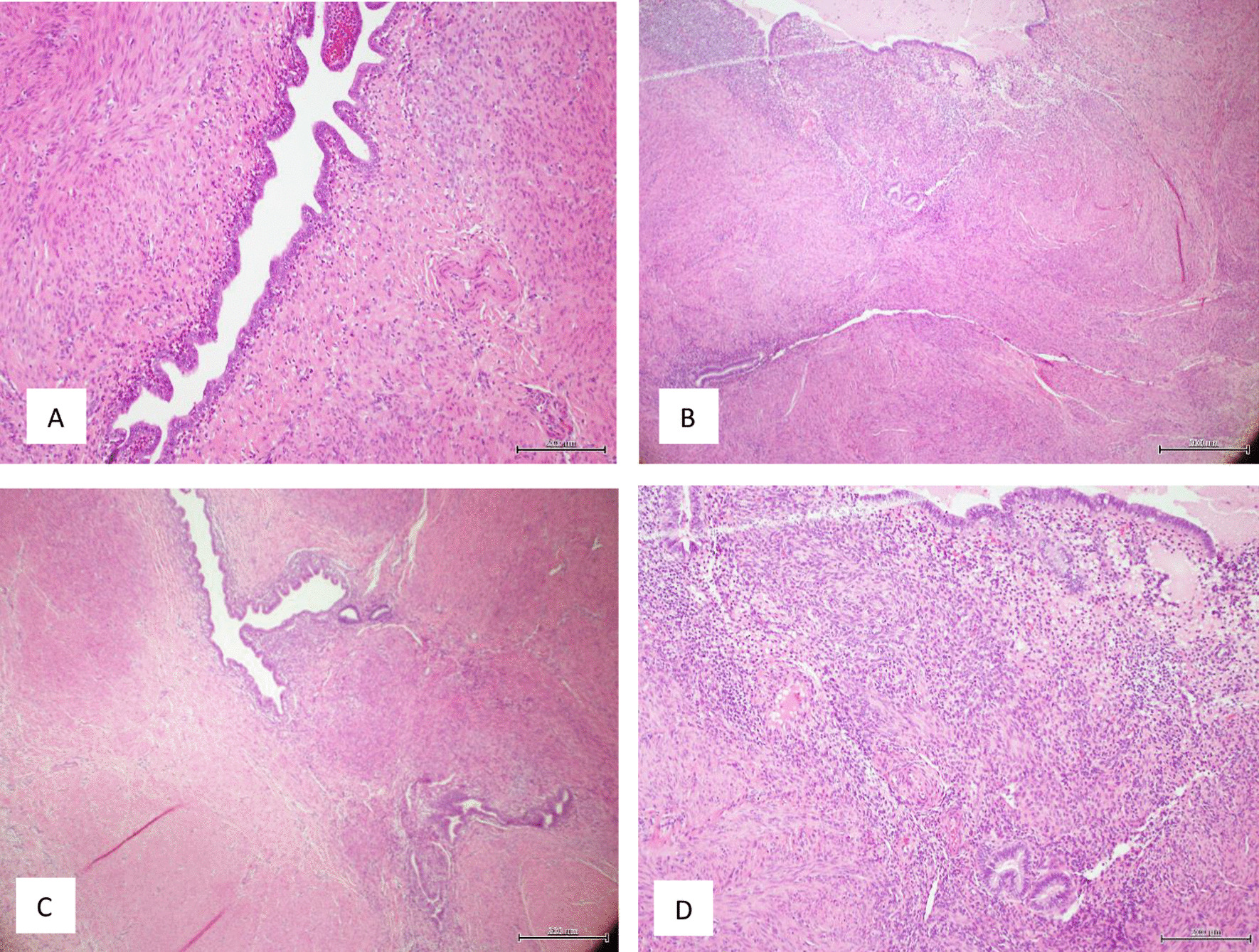
**A** High power view endometrial epithelium and stroma embedded in smooth muscle tissue, **B** endometrial tissue within smooth muscle tissue, **C** multiple foci of adenomyosis, **D** High power view adenomyosis

Her previous supracervical hysterectomy was carried out for a 14-week to 16-week size uterus consisting of fibroids confirmed on histopathology. During the surgical procedure the uterus was morcellated with current evidence of endometrial deposits that were left behind. These residual deposits may have given rise to the endometriotic cysts noted on the latest CT scan. The patient was given Dienogest (progestin) for two months in aims of shrinking the lesion. This is to be monitored with subsequent ultrasound at follow up post completion (Fig. 1). During the course of taking Dienogest, the patient experienced occasional spotting. After the completion of two months course of Dienogest, the ultrasound displayed no change in the endometrial lesion with a finding of a new left-sided lesion that measured 2.5 cm. The patient was managed with Goserelin (GnRH analogue) with add-back therapy for three months thereafter. During the course of treatment, her symptoms of abdominal pain related to the cyst resolved with no evidence of abnormal bleeding. After completion of the three months’ course, a repeat ultrasound was performed which showed reduction in both cysts. The right paraovarian cyst had reduced in measurement from 5.3 × 5 cm to 4.3 × 4 cm. Further plan of management consisted of continuation of 10.8 mg Goserelin (GnRH analogue) injections for another three months. These were injected intramuscularly 3 cm medial to the right anterior iliac spine using an aseptic technique under local anesthetic. In the start of August 2020, a repeat ultrasound three months following the completion of Goserelin revealed a significant increase in the right endometriotic cyst measuring 5.5 × 5.6 cm in comparison to previously 4.3 cm. The left sided cyst displayed minimal change which exhibited resistance to Dienogest and Goserelin. In line with the declining response to medications, the patient was advised for a laparoscopic ovarian cystectomy for which consent was taken and was performed under general anesthesia. During the surgery, some peritoneal adhesions were found involving the anterior abdominal wall, and further extensive adhesions on the left-hand side in the pouch of Douglas involving the bladder secondary to the previous hysterectomy, all which were lysed (Fig. 2). The right mass which was found after great difficulty in the pouch of Douglas, adherent to the sigmoid colon (Fig. 3) was mobilized from the pelvis and separated from the bowel without any evidence of trauma (Fig. 4). A small 1 cm suspected uterine fibroid was identified in the peritoneum overlying the bladder intraoperatively (Fig. 5). This was removed and sent for histology. As for the left ovarian cyst found on ultrasound, it was identified as pockets of peritoneal fluid intraoperatively. Peritoneal cytology was also taken. The usage of a morcellation bag for the fibroid mass revealed its contents to be mainly endometriotic fluid. Due to the patient being thrombocytopenic with a preoperative platelet count of 50 K/uL, 1 unit of platelets was transfused post operatively. There were no unexpected events during the surgery and the estimated blood loss was 400 mL. The surgical pathology report confirmed adenomyosis (Fig. 6) of both specimens, namely the right mass and the initially suspected fibroid. Upon 2 weeks postoperative follow up, the patient was advised to continue treatment with Dienogest instead of Goserelin with further follow up after 6 weeks.

## Discussion and conclusions

We report a case of a 45-year-old woman that was diagnosed with a late presentation of two extrauterine adenomyomas two years after laparoscopic power morcellation at hysterectomy, confirmed by histopathology. Power morcellation is the process of cutting tissue into smaller pieces and has emerged as an advancement in gynecological surgery, making removal of bulky lesions minimally invasive. With small entry points such as those measurable of less than 2 cm in length, removing large tissues through morcellation provides enhanced clinical outcomes such as faster patient recovery, decreased postoperative pain, and respectively fewer wound complications. The process of morcellation consists of inserting a long, tube-like portion of the device into small incisions in the abdomen. A rotating circular blade at the tip allows for the specimen to be drawn out in cylindrical portions without the need for extending the incision. Side effects with power morcellation include: bleeding, infection, bowel obstruction, bruising, oozing or pain at incision site, organ damage, pelvic and abdominal pain, intra-abdominal abscesses, and peritonitis [6]. In addition to this, morcellation is also associated with some rare adverse events, as seen in this patient’s case. After the tissue is morcellated, some residual tissue may be left behind or spread into the abdominal cavity during the removal process. This can allow for implantation to occur and thereby give rise to parasitic fibroids, adenomyotic cells or even endometriosis depending on the origin of the tissue initially morcellated. In the case of an undiagnosed sarcoma, the further spread of such tissue can worsen prognosis and affect survival [7]. For this reason, after morcellation it is important to carefully inspect the abdominal cavity to ensure removal of all tissue fragments or possibly carry out in-bag morcellation to reduce any inadvertent peritoneal spread. Profuse irrigation and suctioning of fluid can remove residual tissue, hence making this highly significant post morcellation [8].

Laparoscopic tissue removal is often carried out with morcellation. This was previously “open” morcellation where tissue was directly removed from the peritoneal cavity with a morcellator. Because of concerns regarding possible seeding of tumor cells in cases of unknown malignancy, newer techniques have developed to reduce these risks by morcellating the tissue in a body or extending all of the port incisions and carrying out morcellation at the wound surface using a scalpel.

With trends of rising cases showing numbers of 1 in 350 having a risk of spreading cancer as opposed to it previously being 1 in 10,000, this data reviewed by Food and Drug Administration (FDA) prompted them to announce their first warning in April 2014 about the risk of laparoscopic power morcellators spreading cancer. In addition, multiple societies released statements in regard to the use of morcellation, which brought forth a variety of queries in aims of progress. The first of many societies was the Society for Gynecologic Oncology (SGO), which suggested evaluation of coexisting uterine or cervical malignancy along with effective communication involving informed and voluntary consent with respect to risks, benefits and alternatives as part of the process of qualifying patients as candidates for morcellation. The American Congress of Obstetricians and Gynecologists (ACOG) presented a statement in aims of conveying the pressing matter around morcellation and their contribution towards tackling it. The American Association of Gynecologic Laparoscopists (AAGL) displayed their stance on morcellation through various actions all governed by striking a balance between acknowledging its advantages as a minimally invasive procedure whilst stating its risk for increased morbidity [9]. The European Society for Gynaecological Endoscopy (ESGE) acknowledges the concern raised regarding morcellation and recognizes the importance of patient education concerning its risks and benefits. Despite the possibility of a presumed fibroid being a sarcoma is quite minimal, the risk of spreading sarcoma cells into the abdominal cavity as a result of morcellation is notable. However, on the other hand the remarkable benefits of power morcellation of fibroids in the realm of minimally invasive surgery counteracts the risks, putting its use to be heavily and consciously weighed. On this matter, the ESGE puts forth a review paper on morcellation of fibroids to shed some light on the discussion. The paper addressed a few clinical questions that could aid surgeons’ decision making of morcellation usage. However, the level of evidence was not sufficient to provide recommendations [10, 11].

Changes implemented by the FDA in accordance with the ongoing updates, consisted of guidance to manufacturers to modify the morcellator product labeling to include contraindications and a boxed warning. These changes were supported by AAGL as well. While some gynecologists became hesitant towards the use of morcellation for liability concerns after the release of FDA warnings; on the other hand, AAGL went forth to encourage improvements towards morcellation and not abandonment in one of their statements [9].

Furthermore, in December 2020, the FDA released an updated safety communication inclusive of the boxed warning and stated, “laparoscopic power morcellators are contraindicated for removal of uterine tissue containing suspected fibroids in patients who are or post-menopausal, or over 50 years of age, or are candidates for en bloc tissue removal” [12]. This encouraged further statements to be released by societies such as ACOG and AAGL exhibiting support towards the role of power morcellation in gynecologic surgery under the condition that strict patient criteria and consent were incorporated [9].

Prospectively, the debate around morcellation truly stems down to two main reflections. The first being the process for evaluation of medical device safety and efficacy. This is relevant as data based on the efficacy of a product can lead to a better understanding about its complications. In the case of morcellation, some pertinent complications would be dissemination of malignancy and port-site metastasis, which can be recognized only after a widespread use and reporting of the product. However, there is an evident lack of centralized reporting of data regarding the usage of a morcellator and its outcomes, all of which has led to the Federal Bureau of Investigation posing queries concerning the transparency of delivering information between manufacturers and consumers [9].

The second reflection is based on the morcellators directly, when comparing with open abdominal procedures. Improved postoperative outcomes, shorter hospital stay and decreased blood loss and surgical site infections are some of strengths of a minimally invasive approach, thereby making it an increasing trend. Assessing the benefits of morcellation vs the theoretical risk of spreading an undetected malignancy is important moving forward, especially until alternatives to morcellation come forth stronger. It is reported that “The risk of dying from an abdominal hysterectomy for fibroids, ranging from 1 in 1,000 to 1 in 2,500, may be equal to or higher than the risk of having an occult malignancy” [9].

With this came room for alternatives which include:

1. Power morcellation in a bag, which allows for the tissue to be contained with minimal intraperitoneal spill.
2. Manual morcellation is another option, where the tissue is cut manually by a scalpel inside the bag.
3. Vaginal hysterectomy where the uterus is taken out through the vagina.
4. Abdominal Hysterectomy & Myomectomy done entirely through an incision directly.
5. En Bloc resection where the uterus, cervix, bladder and partially the rectum is removed in cases of confirmed cancer to avoid spread.

In April 2016, the first containment system to be used with laparoscopic power morcellators was allowed for marketing by the FDA [6]. From there on, with the growing interest in performing contained tissue morcellation within a specimen bag, multiple approaches rose with easy techniques and a minimal change in overall operative time. However, due to the variety of specimen bags available along with numerous surgical techniques used during the process, it has made comparability amongst the containment bags challenging, resulting in difficulty determining superiority of one bag over another. Some drawbacks reported regarding the usage of specimen bags consisted of loss of bag integrity possibly causing leakage of content due to the potential risk of penetrating the bag or in some cases high insufflation pressures within the bag. Nevertheless, ongoing improvements in morcellation devices and containment systems continue to take place in order to achieve optimal goals of being able to remove larger tissue specimens, conserving the containment bag’s integrity and decreasing overall operative time all in order to perform safe morcellation [13].

Estimation of the prevalence of adenomyosis is difficult as it varies from 5 to 70% with a mean frequency of findings at hysterectomy being at roughly 20–30%. The wide range is due to its diagnosis being confirmed at the time of hysterectomies and its variability amongst racial groups [3]. As for the incidence, till date it is still disputed most likely because of different imaging diagnosing criteria [14]. To the best of our knowledge, there have not been many cases documented in the English-literature of Extrauterine adenomyomas, the earliest description dating back to 1981 by Cozzutto et al. [15].

Typically, the extrauterine adenomyomas were found in the pararectal space, ovary and broad ligaments as well as other pelvic sites including paraovarian and parametrial locations [15]. The patient presented in this case report was found to have a suspected fibroid which was later diagnosed as an adenomyoma, however, understanding the general risk of a fibroid having malignant changes influences the intraoperative management of perhaps involving a containment system. Leiomyomas also known as benign uterine fibroids are the commonest pelvic neoplasms to be found in women with an estimated lifetime risk of 70% in Caucasian women and 80% in women of African descent. However, the more worrisome neoplasm that has a poor prognosis is uterine sarcoma which is rarer than leiomyomas as shown by the prevalence of 0.2%. The general consensus is that sarcomas don’t arise from leiomyomas as they usually arise independently and only do so in rare exceptions when leiomyomas display features of atypia and cellular variants [16–18].

In regards to imaging, several studies have shown that both transvaginal ultrasonography (TVUS) and magnetic resonance imaging (MRI) displayed high sensitivities and specificities. TVUS has an estimated pooled sensitivity of 72–82% and pooled specificities of 81–85%. With these numbers reported for TVUS in adenomyosis, it makes it the first line modality in comparison to MRI which has reported sensitivities of between 70 and 93% and specificities between 86 and 93%. Moreover, the differences between the use of MRI and TVUS at diagnosing adenomyosis pertains to a cost difference and accessibility which is in favor of ultrasound, despite it being operator dependent, demanding the operator to have in depth knowledge of uterine anatomy and its cyclic variation. On the other hand, although MRI could provide detailed images that would aid in the detection and diagnosis of adenomyosis, and is less operator dependent, it requires more reader experience at imaging technique optimization to achieve a higher diagnostic accuracy. Computed tomography (CT) is not generally used to diagnose adenomyosis due to its inability to resolve differences between soft tissues which limits its diagnostic capabilities exhibiting poor diagnostic accuracy [4, 19, 20]. This patient however had all three imaging modalities performed, which points towards a point of revision in management as the CT displayed the same findings as the MRI, showing no requirement for the CT scan.

During the surgery, there was difficulty locating the larger mass on the right side although the final location of the mass reflected the description from CT images quite closely. CT and MRI revealed the mass to be an irregular lobulated heterogeneous signal intensity mass seen in the presacral space to the right, located on the right lateral aspect of the recto-sigmoid. The mass measured 4.5 × 4.3 × 4.3 cm in size and exhibited heterogeneous enhancement with areas of non-enhancing cystic component whilst abutting the right piriformis muscle with fluid seen in the pouch of Douglas. Given the detailed description of the mass, ideally it shouldn’t have been very challenging localizing the lesion. Possible factors that made it difficult could be the extensive adhesions she had from her previous surgery. Nonetheless, perhaps having the CT images in the operating theatre to refer back could have aided the localization and may be a point of future changes in management of such cases. The ultrasound images moreover mostly described the size of the lesions over time, with no other descriptive features noted, making it a tool more useful for recording the lesions’ response to treatment.

While the frozen section represents a highly prime purpose, it is only indicated in an intraoperative consultation in the event that the next step of surgery relies on it or if the next immediate step in patient care is to be influenced by it [21]. Moreover, the frozen section analysis has a variable role to play in different scenarios. In the case of ovarian and endometrial tumors, it has a high sensitivity and specificity [22]: whereas, in the case of excluding uterine sarcoma it isn't reliable. This is because for uterine sarcomas, multiple areas need to be sampled in order to establish a diagnosis wherein a frozen section is restricted to a limited tissue sample, making the chances of obtaining a false-negative result highly likely [16]. In relation to our patient’s case, the need for a frozen section is debatable as on one side the patient had elevated serum Ca125 levels (169 U/mL) and a mass that grew in size, while on the other hand, imaging had clearly defined its borders with no other alarming features. Furthermore, the reason why we couldn’t properly identify and diagnose the adenomyosis from the sample obtained by the CT guided fine needle aspiration, was due to the fact that generally its diagnosis depends on its relation to the uterine myometrium, specifically the depth of the myometrial infiltration as well as the number of foci present. And this couldn’t have been achieved in our case due to the adenomyosis being extrauterine. Additionally, the diagnostic criteria is still disputed upon in regards to what is considered as the minimal depth of infiltration and the number of foci present [23, 24].

The modality of treatment of adenomyosis stems from the concept that adenomyosis is an estrogen dependent lesion, such as its kin endometriosis and uterine myomas, as they regress after menopause. Hence rationalizing the use of medications such as Dienogest, a GnRH agonist that induces a hypoestrogenic effect by suppressing the hypothalamic-pituitary-ovarian (HPO) axis. This would consequently help reduce painful symptoms and shrink the size of adenomyotic lesions. Nonetheless, this disruption of the HPO axis will be accompanied by adverse effects such as hot flashes, vaginal dryness and bone mineral loss, that will require the need for add-back therapy if used long term [25, 26]. The patients’ response to GnRH agonists, whether for better or worse in the case of uterine fibroids, could be estimated as early as 4 weeks according to a study by Hackenberg et al. [27]. This study showed a definite 50% reduction in fibroid size is preceded by a 35% reduction after four weeks in 81% of cases [27]. In a study by Matsushima et al. [28] it showed that after hormonal treatment of 16 weeks with GnRH agonist, low-dose estrogen and progestin combination (LEP) and Dienogest in patients with adenomyosis, there was a significant reduction in the GnRH agonist group but there was a non-statistically significant increase in uterine volume in both the LEP and Dienogest groups; where these therapies were ineffective in reducing uterine volume in 53.3% and 44.5% of patients respectively. With greatest increase in size of 2-folds in the LEP and 2.1-fold increase in the Dienogest group, possibly hypothesized to be due to there being four subtypes of adenomyosis when assessed by MRI and each have shown to have different pathogenesis [28]. This reflects upon the treatment course and response the patient had with Goserelin and Dienogest, whereby she eventually gained resistance to them.

In conclusion, in the case of this patient, she presented with the adenomyoma 2 years after laparoscopic power morcellation in hysterectomy, making power morcellation a significant point of concern. While the procedure of morcellation consists of possible surgical complications as seen in this case, these occurrences can be notably reduced when done by a trained surgeon complimented by credible equipment and safe methods. Moreover, advancing interventions such as the containment bags and its various types available aim to decrease these numbers of adverse events all in aims of producing the safest, and minimally invasive outcomes. Ongoing collaboration and efforts between surgeons, medical engineers and manufacturing companies can raise the number of successful outcomes and provide better patient care.

## Data Availability

The datasets used during the current study are available from the corresponding author on reasonable request.

## Abbreviations

CT: Computerized tomography
MRI: Magnetic resonance imaging
HIFU: High intensity focused ultrasound
MRgHIFU: Magnetic resonance imaging high intensity focused ultrasound
USgHIFU: Ultrasound high intensity focused ultrasound
UAE: Uterine artery embolization
FDA: Food and Drug Administration
TVUS: Transvaginal ultrasonography
